# A Report on the Barracks and Hospitals of the United States Army

**Published:** 1871-02

**Authors:** 


### Book Notices.

A Report on the Barracks and Hospitals of the United States Army, with Descriptions of Military Posts. By John S. Billings, Assistant-Surgeon U. S. A. Circular No. 4, War Department, Surgeon-General's Office.

This is a voluminous report of 492 pages, and comprises a large amount of valuable matter, statistics, etc. A considerable space is devoted to the important subject of ventilation. "One objection, it states, has been made to good ventilation, which it is as well to mention, as one of the strongest arguments in its favor. Men will eat more when they have plenty of fresh air than without. Dr. Reid mentions that men in large manufacturing establishments have struck for higher wages where a good system of ventilation had been introduced, as their former wages were insufficient to procure the increased amount of food demanded by their improved appetites. There is little doubt that if some of our barracks were what they should be, in point of air supply, the post food would diminish somewhat."

The statistics given show that of the 151 posts described, "at forty-six the allowance of air space is insufficient, and at only thirty-nine can it be called satisfactory; while even in these last, the ventilation can only be called satisfactory in about half the instances."

In an appendix at the end of the volume are given the results of a number of examinations of the air of barracks made by Assistant-Surgeon V. B. Hubbard for the purpose of ascertaining the amount of carbonic acid and of organic matter present at different times. In regard to the examinations, he says: "The perception by the sense of smell of the presence of organic matter is the usual and the standard test of the fact of insufficient ventilation. For the simple fact of good or bad ventilation, the accuracy of the test is greatly influenced by the temperature, and it is sufficiently delicate only in a tolerably warm room. When we attempt to determine the amount of organic emanations from the body present in a given space, we find that they are so small in actual mass, and of so complex and so indefinite a character, that they evade to a certain extent the powers of chemical titration.

The measurement of the carbonic acid which is added to the air by animal respiration is much freer from the difficulties above alluded to; the quantity given off is more considerable, as air, in passing through the lungs, has its carbonic acid increased about one hundred times, or from about four parts in ten thousand to four parts in one hundred. Moreover, the chemical affinities of carbonic acid, although comparatively feeble, are well defined, and it is capable of tolerably exact chemical estimation."

From these examinations it was determined that the amount of air which writers on hygiene have held to be the minimum supply consistent with perfect healthfulness, *viz.*, 2,000 cubic feet per man, per hour, was, at the posts visited, attained only in exceptional instances; but that in most cases an extension and slight modification of the existing systems of ventilation would probably give a sufficient circulation of air.

Among the valuable statistics contained in the report are extensive tables showing the ratio of total mortality (exclusive of deaths from epidemics, accidents, and wounds) due to various classes of diseases in the United States army and navy, the English army and navy, the French army and navy, the Italian army, and in civil life; also, tables showing the comparative prevalence of the principal diseases in the different departments of the army.

Accompanying the report is Circular No. 3, a small volume, containing approved plans and specifications for post hospitals. These reports are a valuable addition to our literature.

